# Probiotic *Lactobacillus rhamnosus* GR-1 and *Lactobacillus reuteri* RC-14 May Help Downregulate TNF-Alpha, IL-6, IL-8, IL-10 and IL-12 (p70) in the Neurogenic Bladder of Spinal Cord Injured Patient with Urinary Tract Infections: A Two-Case Study

**DOI:** 10.1155/2009/680363

**Published:** 2009-06-04

**Authors:** Kingsley C. Anukam, Keith Hayes, Kelly Summers, Gregor Reid

**Affiliations:** ^1^Canadian Research and Development Centre for Probiotics, Lawson Health Research Institute, London, ON, Canada N6A 4V2; ^2^Department of Microbiology and Immunology, University of Western Ontario, London, ON, Canada N6A 3K7; ^3^Department of Physical Medicine and Rehabilitation, University of Western Ontario, London, ON, Canada N6A 3K7

## Abstract

The management of urinary tract infection (UTI) in individuals with spinal cord injury (SCI) continues to be of concern, due to complications that can occur. An emerging concept that is a common underlying pathophysiological process is involved, wherein pathogens causing UTI have a role in inflammatory progression. We hypothesized that members of the commensal flora, such as lactobacilli, may counter this reaction through anti-inflammatory mediation. This was assessed in a pilot two-patient study in which probiotic *Lactobacillus rhamnosus* GR-1 and *Lactobacillus reuteri* were administered to one patient and placebo to another, both along with antibiotics to treat acute UTI. Urinary TNF-alpha was significantly downregulated (*P* = .015) in the patient who received the probiotic and who used intermittent catheterization compared with patient on placebo and using an indwelling catheter. The extent to which this alteration resulted in improved well-being in spinal cord injured patients remains to be determined in a larger study.

## 1. Introduction

Chronic urinary tract infection (UTI) and bladder carcinoma remain prominent complications and causes of morbidity and mortality following spinal cord injury (SCI) [1–3]. An emerging concept is that there is a common underlying pathophysiological process involved, wherein pathogens causing chronic UTI induce inflammation and other effects, for example which increase the risk of bladder cancer [3]. Chronic UTI leads to earlier mortality and although the mechanisms are unclear, the production of carcinogens and inflammatory factors in the bladder has the potential to cause cancer at local and distant tissue sites.

Innovative ways to modulate inflammatory processes are needed, as current neurogenic bladder management relies almost totally on antibiotic use, and this is essentially ineffective in eradicating multidrug resistant bacteria in dense biofilms. Previous studies have shown that probiotic bacteria have the potential to interfere with infectious and inflammatory processes, including in the urogenital tract [4–7]. The oral intake of probiotic strains *Lactobacillus rhamnosus* GR-1 with *Lactobacillus reuteri * (formerly *fermentum*) RC-14 has been shown to reduce the pathogen load in the urogenital tract [7], and a recent study using a *L. acidophilus * strain showed equal efficacy to prophylactic antibiotics in preventing recurrent UTI in children [8].

Direct instillation of lactobacilli into the bladder of patients with neurogenic disease has been attempted without success, with the lactobacilli being not able to colonize the bladder [9]. An avirulent *E. coli* strain has been implanted into the bladder of SCI patients and shown to reduce the ability of infectious organisms to colonize [10]. A study showing that probiotic lactobacilli can be given with antibiotics to cure acute infection [7], and oral administration of lactobacilli can mediate analgesic functions in the gut [11] formed a basis for the present protocol. In addition, the reported anti-inflammatory properties of *L. rhamnosus* GR-1 and *L. reuteri * RC-14 [12] made it possible to explore their effect on inflammation in SCI patients. As a proof-of principle study, two SCI patients with UTI were administered antibiotics plus lactobacilli or placebo.

## 2. Patient Details and Experimental Methods

### 2.1. Patient Recruitment

The subjects were recruited from the outpatient population and “alumni” of the Regional SCI Rehabilitation Programs in London, Ontario (Parkwood Hospital/St Joseph's Health Care).

### 2.2. Study Procedures

After obtaining signed informed consent (Day 0), a blood sample was collected for cytokine, liver and kidney marker analysis, and a urine sample was collected to confirm infection and to examine cytokine levels. The subjects were treated with antibiotics (as detailed below), randomized to receive the probiotics/placebo capsules and instructed on how and when to take each (two capsules taken 1 hour after dose of antibiotic). Both subjects came for visit 2, (midway through antibiotic treatment), visit 3 (at the end of antibiotic treatment), visit 4 (day 30) and visit 5 (day 90). At these visits, the Coordinator/Nurse collected blood and urine samples for cytokines analysis.

### 2.3. Method for Analyzing Urine and Serum Cytokines—Biomultiplex Panels (Premixed Assays)

Cytokine concentrations from urine and serum were analyzed using fluorescent microsphere-based multiplexing technology with Bio-Plex 200 analyzer (Bio-Rad, Inc., Calif, USA). Cytokines, interleukin (IL)-6, IL-8, IL-10, IL-12(p70), and tumor necrosis factor (TNF)-*α* were analyzed using a premixed multiplex panel (Bio-Rad, San Diego, CA). All determinations were performed in duplicate wells and concentrations calculated by the Bio-Plex Manager software, using the standard curves derived from the recombinant cytokine standards.

### 2.4. Patient 1

A 31-year-old female sustained a complete spinal cord injury with ASIA score of S4–S5 on the 13 May, 2003. She has urinary incontinence and no urethral sensation. She has a history of recurrent UTI, each treated with antibiotics. She reported no pain in her bladder or kidney, and uses an indwelling catheter to manage micturition. She visited her physician at Parkwood hospital on 11 October, 2007, for a suspected UTI. Her urine sample was slightly cloudy, positive for nitrite, and moderate for leukocyte esterase. The culture yielded *Citrobacter freundii* (>100 × 10_6_ cfu/L) and *Enterococcus spp* (10–100 × 10_6_cfu/L). The *Citrobacter freundii* was susceptible to Cefotaxime, Ciprofloxacin, Gentamicin, Imipenem, Pipercillin/Tazobactam, Tobramycin, and Trimethoprim/Sulfamethoxazole. *Enterococcus spp* was susceptible to Ampicillin, Ciprofloxacin, and Nitrofurantoin. On screening for inclusion in the study, the subject weighed 63.6 kg, had a blood pressure of (Systolic/Diastolic) 96/72 mmHg, sitting pulse rate of 80 beats/minute, and her means of mobility was a wheelchair. She was prescribed oral Ciprofloxacin (500 mg × 2) daily for 12 days and the study product (two capsules daily for 90 days).

### 2.5. Patient 2

A 45-year-old male with a medical history of incomplete spinal cord injury in 1999 with ASIA score of muscle grade <3 had more than 24 treated episodes of UTI since then (treated with Norfloxacin, Amoxycillin, Ciprofloxacin, Cotrimoxazole, Macrobid, Cefexime). His last antibiotic treatment for a UTI was on 29 October, 2007. On 22 November 2007, he had signs and symptoms synonymous with UTI and decided to take his urine sample to the laboratory for culture and sensitivity. The subject had heard about the probiotic study, and traveled with his caregiver more than 293 kilometers to the study center at Parkwood hospital, London, Ontario, where he was screened and enrolled on 29th November, 2007. The subject used a wheelchair and at enrolment weighed 95.6 kg, with a blood pressure of 120/72 mmHg, sitting pulse of 89 beats/minute, respiration of 18 breaths/minute and a body temperature of 37.3°C. He used intermittent catheterization for bladder management. He provided a urine sample (30 mls) for urinalysis (dipstick) and cytokine assessment. A blood sample (10 mls) was collected for serum cytokines. The urinalysis result showed presence of moderate leucocytes, positive nitrite, cloudy a strong urine odor, and *Escherichia coli* (>100 × 10_6_ cfu/L), sensitive to Cephalothin, Gentamicin, Ciprofloxacin, Cotrimoxazole, Cefixime, Ampicillin, and Nitrofurantoin. The subject was prescribed Nitrofurantoin 100 mg for 14 days and randomized to receive 2 capsules of probiotic lactobacilli daily for 90 days.

### 2.6. Statistical Analysis

Mann-Whitney U test was used when comparing the means of continuous data between two independent groups. Two-sided Fisher's Exact Test was used for significant associations between two categorical variables in 2 by 2 contingency tables. Differences were considered statistically significant if *P*-value was <.05.

### 2.7. Ethics Review Board

The ethics review board of the University of Western Ontario and Health Canada gave approval for this study.

## 3. Results

**Figure 1:**
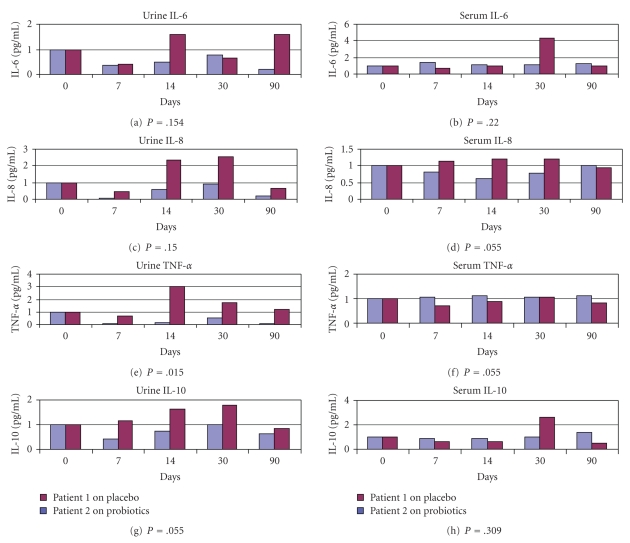
*Comparative urine and serum cytokines from patient 1 and patient 2.* Serum and urine samples were collected from the patients at days 0, 7, 14, 30 and 90. Interleukins (IL)-6, IL-8, IL-10, IL-12(p70), and tumor necrosis factor (TNF)-*α* were analyzed using a premixed multiplex panel and concentrations calculated by the Bio-Plex Manager software. Differences were considered statistically significant if *P*-value was <.05.

Urine and serum cytokine results for patient 1 (Placebo) and patient 2 (Probiotics) for Visit no. 1 (day 0) to visit no. 5 (day 90) are shown in Figure 1. At baseline (day 0) the cytokine value was taken as a ratio, and subsequent visits were calculated as a ratio to the baseline cytokine value.

The cytokines from patient 2 were downregulated compared initial baseline presentation with UTI. For example his IL-8 baseline value was 545.63 pg/mL at inclusion, downregulated to 36.74 pg/mL at day 7 amounting to 93.3% reduction, compared to a 56.9% reduction in patient 1. There was a slight increase in urinary cytokines for patient 2 at day 30 but the values especially IL-6, IL-8, IL-12 and TNF-alpha did not go above the baseline values obtained ab initio when he had UTI. The urinary cytokine level for IL-6 was 0.508 pg/mL on day 14 compared to 1.581 pg/mL for the placebo patient 1, and 0.223 pg/mL on day 90 compared to 1.58 pg/mL. 

For urinary IL-8, there was downregulation in patient 2 in all sample days (0.067 pg/mL, 0.607 pg/mL, 0.941 pg/mL, and 0.228 pg/mL) compared to patient 1 (0.432 pg/mL, 2.375 pg/mL, 2.556 pg/mL, and 0.662 pg/mL) (*P* = .15). The serum IL-8 had a similar trend with a significant difference (*P* = .05) between patients 1 and 2.

Urinary TNF-alpha was significantly downregulated in patient 2 (*P* = .015) on all the days sampled, compared with patient 1: patient 2 had TNF-alpha on day 7 (0.08 pg/mL), day 14 (0.13 pg/mL), day 30 (0.52 pg/mL) and 90 (0.106 pg/mL), while patient 1 had 0.68 pg/mL, 3.01 pg/mL, 1.75 pg/mL, and 1.18 pg/mL. For serum TNF-alpha, there was no significant difference (*P* = .055) between the two patients.

Patient 2 had urinary and serum IL-10 regulated but this was not statistically different from patient 1 (*P* = .055 for urine and *P* = .309 for serum). Urinary IL-12 (p70) was also downregulated in patient 2 on all sampling days compared to patient 1 (0.552 pg/mL versus 0.808 pg/mL, 0.884 pg/mL versus 1.808 pg/mL, 0.884 pg/mL versus 1.178 pg/mL, and 0.552 pg/mL versus 0.84 pg/mL) (*P* = .20). Serum IL-12 (p70) was not detected in either patient.

## 4. Discussion

This is the first study showing the effect of probiotics in augmenting antimicrobial therapy in downregulation of inflammatory urinary cytokines in a spinal cord injured individual with UTI. Downregulation of proinflammatory cytokines, especially IL-6, IL-8 and TNF-alpha, in the patient who took probiotics is important as these cytokines contribute to an immune-mediated impairment of axonal conduction [13, 14]. TNF-*α* is also strongly implicated in neuropathic pain. It induces axonal damage, macrophage recruitment, and ectopic activity in peripheral nerve fibers [15]. 

In men, UTI is invariable due to urethral catheter ascension of pathogens [16]. In females, lactobacilli therapy leads to the probiotic organisms reaching the vagina and potentially the urethra via natural ascension along the perineal skin. However, this would not occur in males. Thus, physical competitive exclusion of the uropathogens is unlikely the mechanism of any positive anti-infective effects of the lactobacilli. However, lactobacilli can modulate mucosal immunity in the gut and even apparently reduce the risk of bladder cancer recurrence via this pathway [4]. In patient 2, anti-inflammatory effects were noted in the serum as well as the urine thereby potentially supporting this mechanistic route. In acute UTI, IL-6 and TNF-alpha are particularly associated with symptomatology [17, 18], and the ability of probiotic lactobacilli to downregulate these cytokines could have implications for better managing UTI in SCI as well as non-SCI patients. 

A previous study found that consumption of products containing *L. gasseri* CECT 5714 and *L. coryniformis* CECT 5711 induced an increase in the proportion of serum natural killer (NK) cells and in IgA concentrations [19], providing additional precedent for our findings. 

IL-12 (p70) was only detected in the urine of patients with lower values observed in the probiotics patient. It is noteworthy that human Interleukin-12 (p70), also known as natural killer cell stimulatory factor (NKSF) or cytotoxic lymphocyte maturation factor (CLMF), is a potent regulator of cell-mediated immune responses. IL-12 also promotes the development of proinflammatory-Th1-like CD4_+_ cells and cytotoxic CD8_+_T cells and it induces NK and T cells to secrete large amounts of the proinflammatory cytokine [20].

It could be argued that the pathogenesis of gender-related differences in the rate of infectious diseases may have played a role between the two patients but interestingly under physiological conditions, the previous studies have shown no significant gender-related difference in the level of urine cytokines especially IL-6, IL-10, TNF-alpha [21]. Urine proinflammatory cytokines IL-6 and IL-8 have been found to be significantly higher in male patients with bacteriuria than in males without bacteriuria. It is also possible that patient 1 with an indwelling catheter presents a different inflammatory scenario than patient 2 who used intermittent catheterization. Such factors should be explored when a larger study is designed. 

In summary, this two-case study provided valuable insight into how probiotic lactobacilli could be tested to determine if they provide adjunct benefits in the management of UTI in SCI patients. The preliminary evidence of immune modulation in a male patient also suggests that such therapy could have applicability to the care of males with UTI, a finding that has not been explored in the previous probiotic studies.
